# CONSIDERATIONS FOR CROSS-NATIONAL APPROACHES TO ESTIMATING PREVALENCE OF DEMENTIA

**DOI:** 10.1093/geroni/igae098.2082

**Published:** 2024-12-31

**Authors:** Wenjie Cai, Richard Jones, Alden Gross

**Affiliations:** Johns Hopkins University, Baltimore, Maryland, United States; Brown University, Providence, Rhode Island, United States; Johns Hopkins Bloomberg School of Public Health, Baltimore, Maryland, United States

## Abstract

Comparing dementia prevalence across countries and contexts is crucial for effective public health planning. The Harmonized Cognitive Assessment Protocol Project (HCAP) is a global effort to characterize dementia risk using population-representative samples. Using the HCAP studies from the US, England, and India, we evaluated variations of a classification algorithm based on neuropsychological norms. Variations of the algorithm included variables used to define a robust normative sample (e.g., depression, stroke history, previous diagnosis of dementia, basic and instrumental activities of daily living), characteristics adjusted for when standardizing cognition in the normative sample (e.g., age, sex, education, race/ethnicity, literacy), numbers and types of cognitive domains, and the choice of cut-offs of the components in classification algorithm such as cognitive scores, self-rated memory, and informant-rated impairment. Across each country, we varied these parameters. The overall prevalence of dementia ranged from 8% (India) to 9% (US, England). As expected, using a more liberal 1.0 standard deviation (SD) cutoff on cognitive tests rather than 1.5 SD led to higher dementia prevalence by 3-7%. Excluding orientation to time/place from the cognitive domains resulted in lower prevalence of dementia by 2-3% across countries but a markedly lower prevalence of Mild Cognitive Impairment (by 10% or more), whereas less restrictive criteria for defining a normative sample resulted in slightly lower prevalence of dementia (by 1-2%). This study underscores the importance of consistent methodologies for cross-national dementia prevalence estimates, and also informs the relative importance of certain modifications to criteria.

